# Revisiting Activity of Some Nocodazole Analogues as a Potential Anticancer Drugs Using Molecular Docking and DFT Calculations

**DOI:** 10.3389/fchem.2021.628398

**Published:** 2021-03-24

**Authors:** Muhammad Khattab, Ahmed A. Al‐Karmalawy

**Affiliations:** ^1^ Department of Chemistry of Natural and Microbial Products, Division of Pharmaceutical and Drug Industries, National Research Centre, Cairo, Egypt; ^2^ Department of Pharmaceutical Medicinal Chemistry, Faculty of Pharmacy, Horus University-Egypt, New Damietta, Egypt

**Keywords:** anthelminthic, anticancer, tubulin inhibitors, molecular docking, DFT calcualtions, drug repurposing

## Abstract

Although potential anticancer activities of benzimidazole-based anthelmintic drugs have been approved by preclinical and clinical studies, modes of binding interactions have not been reported so far. Therefore, in this study, we aimed to propose binding interactions of some benzimidazole-based anthelmintics with one of the most important cancer targets (Tubulin protein). Studied drugs were selected based on their structural similarity with the cocrystallized ligand (Nocodazole) with tubulin protein. Quantum mechanics calculations were also employed for characterization of electronic configuration of studied drugs at the atomic and molecular level. Order of binding affinities of tested benzimidazole drugs toward colchicine binding site on tubulin protein is as follows: Flubendazole > Oxfendazole > Nocodazole > Mebendazole > Albendazole > Oxibendazole > Fenbendazole > Ciclobendazole > Thiabendazole > Bendazole. By analyzing binding mode and hydrogen bond length between the nine studied benzimidazole drugs and colchicine binding site, Flubendazole was found to bind more efficiently with tubulin protein than other benzimidazole derivatives. The quantum mechanics studies showed that the electron density of HOMO of Flubendazole and Mebendazole together with their MEP map are quite similar to that of Nocodazole which is also consistent with the calculated binding affinities. Our study has ramifications for considering the repurposing of Flubendazole as a promising anticancer candidate.

## Introduction

Microtubules play a key role in the invasion and metastatic spread of tumor cells, depending on its crucial roles in mitosis, signaling, trafficking, proliferation, and migration of eukaryotic cells (Honore, Pasquier and Braguer, 2005). Drugs targeting microtubular proteins constitute a major and promising anticancer drug category exhibiting both antimitotic and antiangiogenic properties, besides inhibiting tumor progression in cancer and endothelial cells (D Katsetos and Draber, 2012). Colchicine binding site (CBS) is one of the five important identified binding sites on tubulin protein with the longest history of research as an anticancer target (Wang and Zhang, 2016).

The success rate for new anticancer drugs from Phase I trial to commercial use by FDA approval is estimated to be around 6.7% from 2003 to 2011, taking about 8.3 years as an average (Hay et al., 2014; Takebe, Imai and Ono, 2018). The overall numbers of cancer deaths massively grow, rendering it the leading cause of death across all age groups by 2020 (Weir et al., 2016). Therefore, there is a great global need for rapidly approved and effective anticancer drug candidates.

Computational drug repurposing, a new area of drug repurposing, has been intensively developed due to breakthrough advances in fields of molecular, genomic and phenotypic data of pharmacological compounds (Park, 2019). Drug repurposing is an accelerated tool for drug development by seeking new indications for already approved drugs rather than discovering *de novo* drug compounds and constitutes nowadays 30% of the newly marked drugs in the United States (Pushpakom et al., 2018; Park, 2019) Many successful repurposed drugs have been introduced by FDA as in case of Aspirin, used for the treatment of stroke and/or myocardial infarction, Topiramate, used for the treatment of obesity, and Mifepristone, used for the treatment of hyperglycaemia in Cushing’s syndrome (Polamreddy and Gattu, 2019).

Benzimidazole nucleus is a pharmacophore in lots of bioactive heterocyclic compounds with a wide range of biological and clinical applications. Moreover, benzimidazole derivatives constitute the isosteric structures of many naturally occurring nucleotides, which allows interacting easily with the living system biopolymers (Narasimhan et al., 2012). As a result, it constitutes a large group of drugs exhibiting various therapeutic activities such as anthelmintic, antiviral, antihypertensive, antioxidant, and anticancer (Yadav and Ganguly, 2015). Benzimidazole anthelmintic drugs are widely used due to their low toxicity and high efficiency against a wide range of helminth species (Köhler, 2001). Their mechanism of action is based on specific binding to tubulin subunit of microtubular protein, which results in a disruption of its structure and function (Lacey, 1990).

Based on the aforementioned data, benzimidazole derivatives became an attractive target for drug repurposing trials. The cytotoxic studies on benzimidazole-based drugs revealed their potential activity as colchicine binding site inhibitors (CBSIs) (Wang and Zhang, 2016; Al-Karmalawy and Khattab, 2020). Despite none of the benzimidazole-based drugs have been granted food and drug administration (FDA) approval for targeting the colchicine binding site on tubulin till now, the preclinical and clinical studies revealed the CBSIs are less susceptible to drug resistance development rendering them a potential target for cancer treatment (McLoughlin and O’Boyle, 2020).

Benzimidazole anthelmintic drugs having over 40 years of safe use as over the counter medications (Geary et al., 2010). They are meeting many characteristic features to be desirable for repurposing such as well-known safety profiles, well-described pharmacokinetic studies, and low prices (Biodegradabilních and Konjugátů, 2015). *In vitro* and *in vivo* studies revealed the potential of some members of benzimidazole anthelmintic drugs to suppress tumor progress through inhibition of multiple biological targets such as tubulin polymerization and angiogenesis (Mukhopadhyay et al., 2002; Králová et al., 2013; Raghunath and Viswanathan, 2014; Guerini et al., 2019).

**FIGURE 1:**
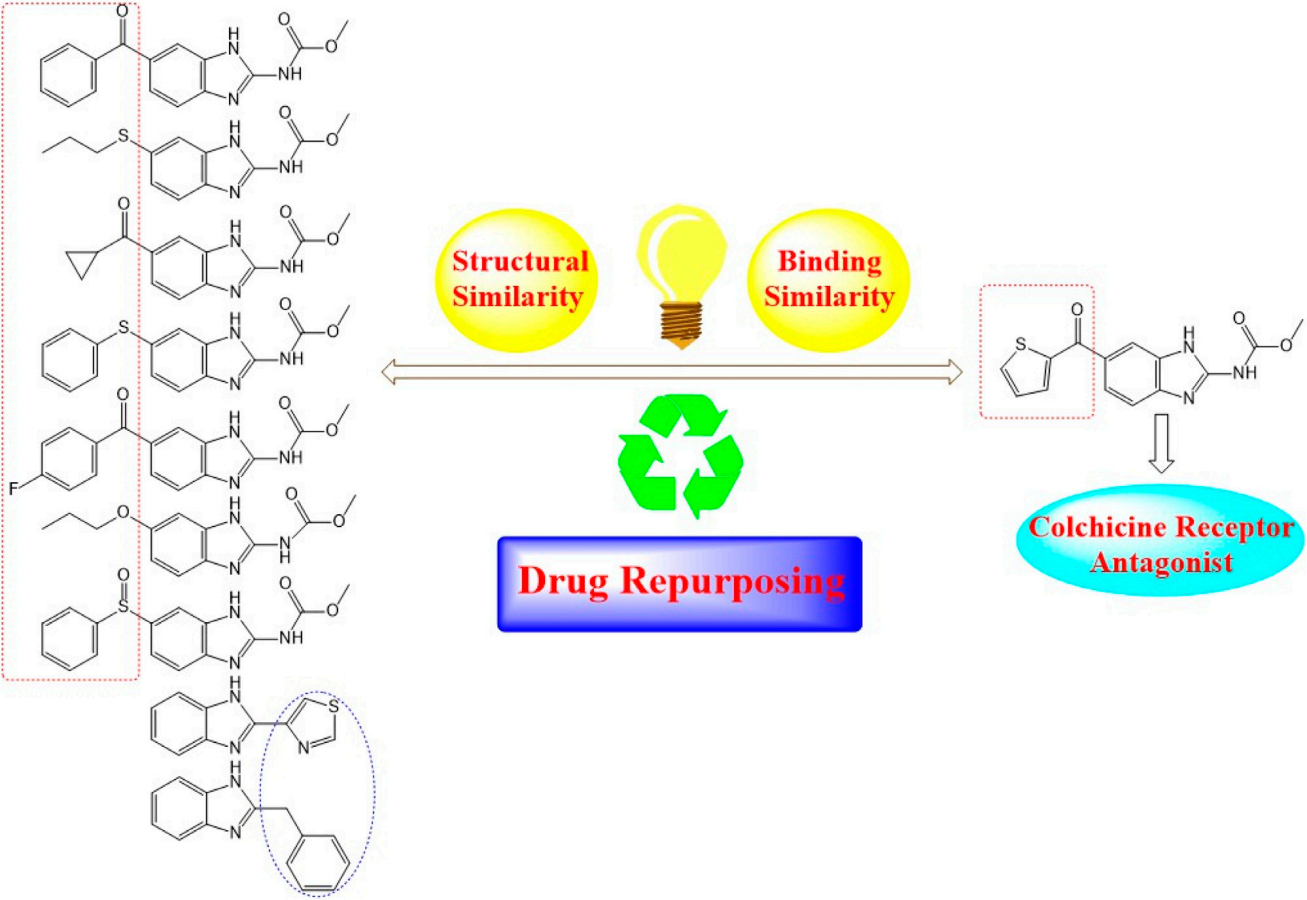
Diagram illustrating structural similarity between benzimidazole anthelmintic drugs and NZO (reference drug).

Nocodazole (NZO) is a benzimidazole-based experimental drug targeting both protein kinases and microtubules. It is used as a lead compound for the discovery of novel CBSIs (Geary et al., 2010). In this manuscript, we propose modes of binding interactions between nine benzimidazole-based drugs in comparison with the reference cocrystallized NZO drug (Figure 1) at colchicine binding site of tubulin protein. The first seven members of the nine elected benzimidazole anthelmintic drugs (1–7) exhibit structural similarity (benzimidazole core and carbamate moiety) with NZO. Studies reported here is a continuation to our previous work on NZO and a benzimidazole-based anthelmintic drug (Mebendazole) (Al-Karmalawy and Khattab, 2020; Khattab, 2020).

## Methodology

### Docking Studies

#### Tested Compounds Optimization

Docking studies using the Molecular Operating Environment (MOE) software package (Inc., 2016) were performed to evaluate the activities and binding modes of nine benzimidazole anthelmintic drugs compared to NZO complexed with CBS (Wang and Zhang, 2016). Mebendazole (1), Albendazole (2), Ciclobendazole (3), Fenbendazole (4), Flubendazole (5), Oxibendazole (6), Oxfendazole (7), Thiabendazole (8) and Bendazole (9) were downloaded from the PubChem website (https://pubchem.ncbi.nlm.nih.gov/). The nine tested compounds underwent energy minimization after examining the structure and the formal charges on atoms using a 2D depiction model. The partial charges were also automatically calculated. Structure of the co-crystallized NZO obtained from two subunits of the target tubulin protein (B and D subunits) and the nine tested benzimidazole drugs were imported together in the same database and saved in the form of MDB file to be docked in two separate processes for each subunit pocket, B and D, respectively.

#### Target Tubulin Active Site Optimization

The X-ray structure for tubulin protein (PDB: 5CA1) complexed with the native NZO was obtained from the Protein Data Bank (http://www.rcsb.org/). All steps for the preparation of the target protein for docking calculations were done. The addition of hydrogen atoms with their standard 3D geometry to the system, automatic correction to check for any errors in the connections and types of atoms, and fixation of the potential of the receptor were also performed. The selection of the same active site of co-crystallized inhibitor in the protein structure was done by using Site Finder where dummy atoms were created at the same binding site of the pocket.

#### Docking of Molecules into Colchicine Binding Site of Tubulin

Docking of the database composed of the nine benzimidazole drugs together with NZO was performed. The following methodology was applied: the prepared protein active site file was loaded, and then the general and template docking processes using dock tools were initiated. Dummy atoms act as the docking site, triangle matcher is the placement methodology, London dG is the scoring methodology, rigid receptor represents the refinement methodology, and GBVI/WSA dG is the scoring methodology for selection of the best 10 poses from 100 poses for each compound. After completion of docking processes, the obtained poses were studied, and the best ones showing the best interactions of ligand–colchicine binding site of tubulin were selected and studied accordingly.

### Molecular Mechanics Calculations

Density functional theory (DFT) based Becke’s three-parameters Lee-Yang-Parr hybrid functional (B3LYP) (Becke, 1993; Becke, 2001) was employed in the calculations. The geometry of all compounds was initially optimized using the B3LYP/3-21G model and reoptimized using the B3LYP/6-31G model and B3LYP/6-311+G* model. No imaginary frequencies were obtained from the optimized structures demonstrating that the corresponding geometries are true local minima. Conductor-like polarizable continuum model (CPCM) (Cossi et al., 2003) and dielectric constant of solvent water (*ε* = 78.35) were used to approximately describe the polarity of colchicine binding pocket. All simulations were performed using GAUSSIAN 09 Revision C.01 (Frisch et al., 2016) on Swinburne supercomputing facilities.

## Results and Discussion

Crystallographic data of co-crystallized NZO complexed with tubulin protein (PDB: 5CA1) revealed the existence of two binding sites for NZO inside the protein, subunits B and D, respectively. It was obvious that subunit D is larger than subunit B in size. By analyzing the binding modes (3D graphic views) of NZO inside the two subunits (Table 1), it was found that NZO exhibits almost the same binding interactions within protein subunits B and D, where three H-bonds were observed between Glu198 and NH-carbamate (one H-bond) and NH(3)-benzimidazole (two H-bonds). Another H-bond was found between Val236 and the protonated NH(1)-benzimidazole. Besides, NZO docked in subunit B was able to form H-bond between CO of carbamate moiety and Asn165. Moreover, a hydrophobic interaction was found between Ala314 and the thiophene ring. In the case of subunit D, two additional H-bonds were observed between NZO and Cys239, which were a direct H-bond with the oxygen atom of the carbonyl group and an indirect H-bond with the carbonyl oxygen through the bridging water molecule (H_2_O616). Hydrophobic interactions were also observed between Leu253 and the benzene and imidazole moieties. The 2D graphic views of binding interactions between studied benzimidazole ligands and NZO with the target protein are deposited in Supplementary Material.

**TABLE 1:** The 3D view of binding interactions between tested benzimidazole drugs and NZO-binding pocket within Tubulin subunit B and D (PDB: 5CA1) beside the solved NZO complex (Native) and the docked complex (Docked). Red dashed lines refer to hydrogen bonds, while the black ones denote hydrophobic interactions.

Drug	Protein (5CA1) subunit B	Protein (5CA1) subunit D
NZO (native)	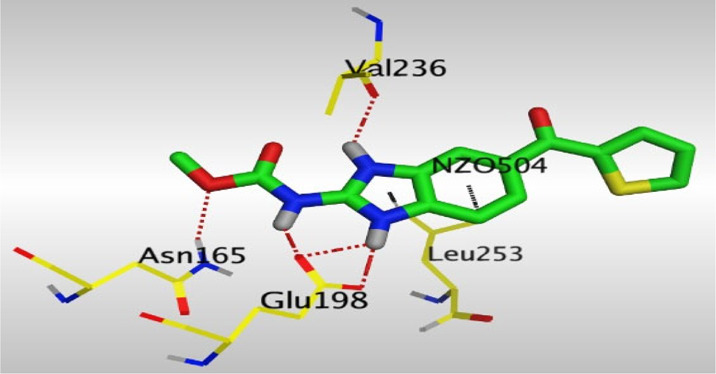	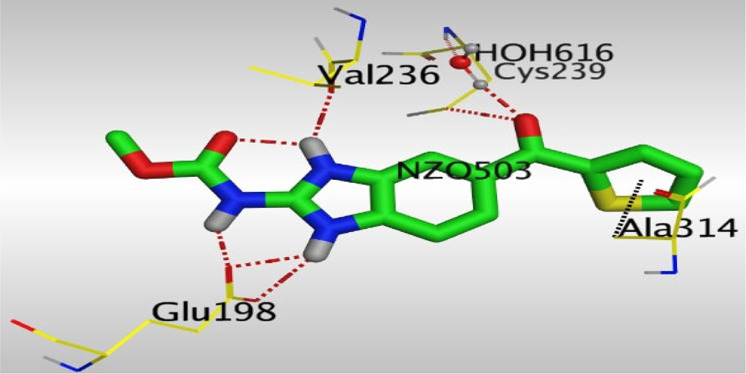
NZO (docked)	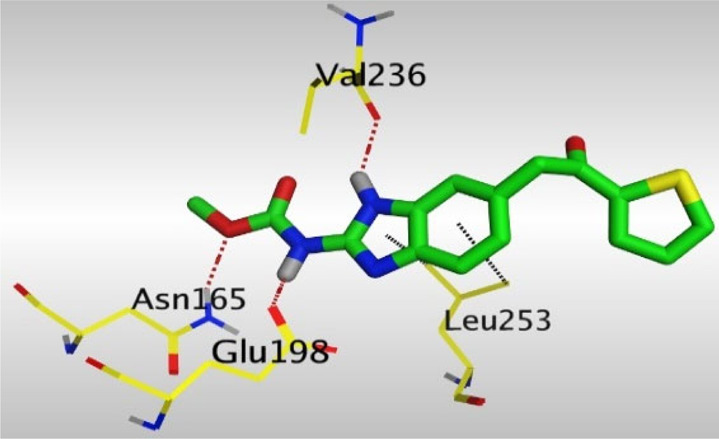	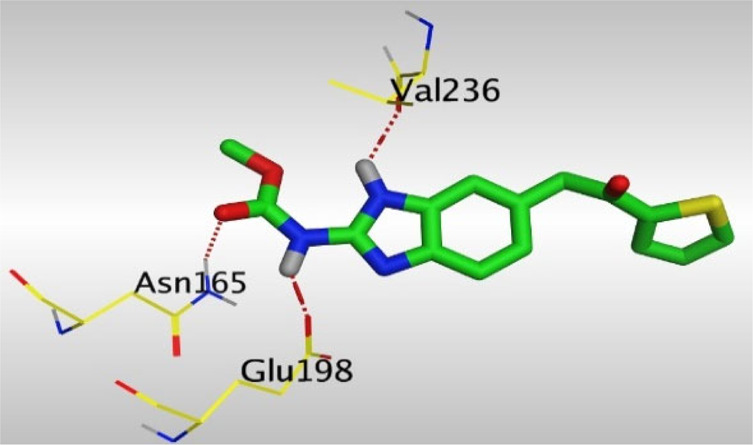
Mebendazole	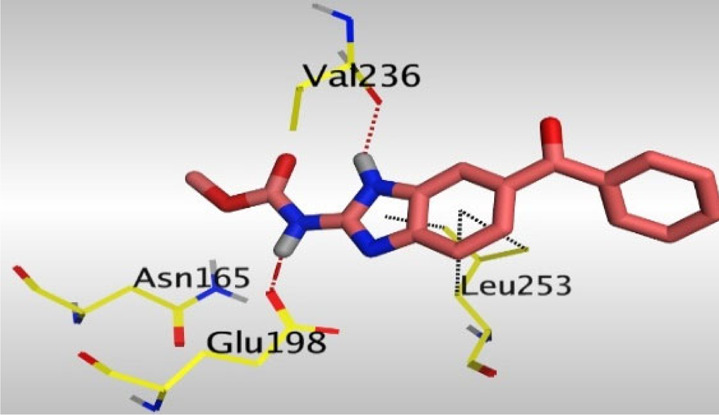	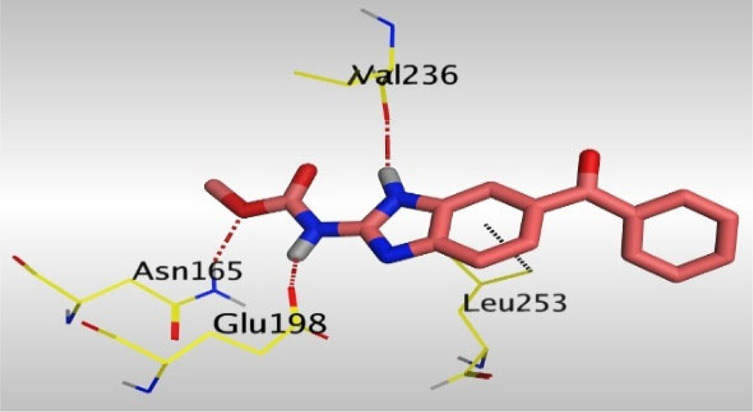
Albendazole	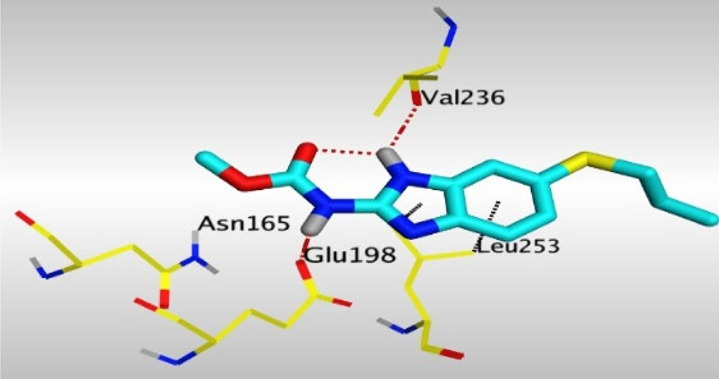	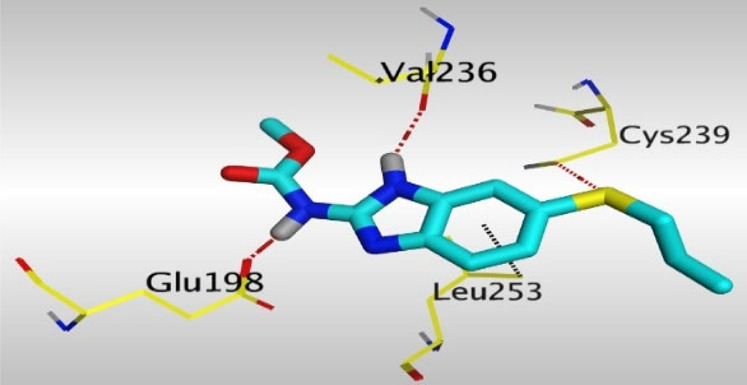
Ciclobendazole	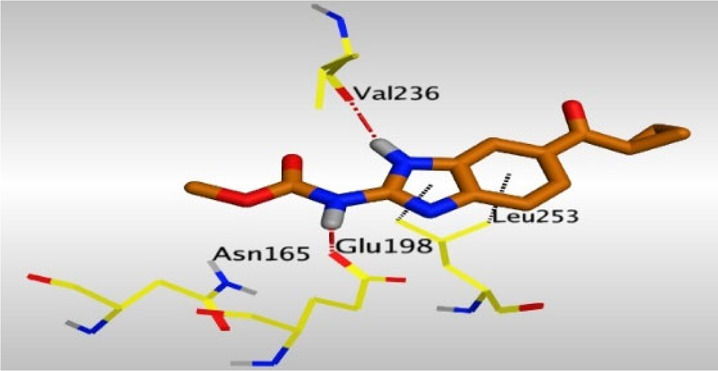	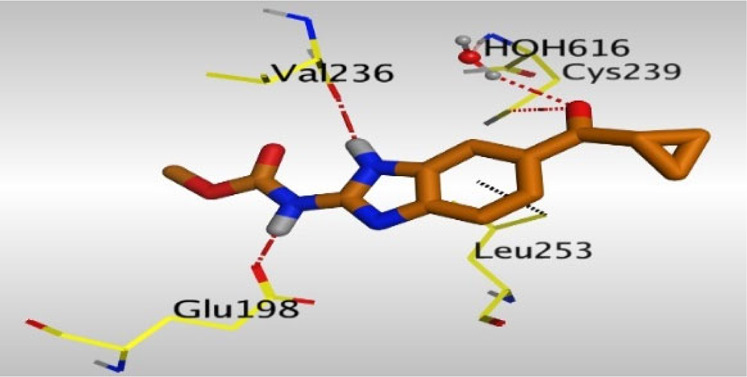
Fenbendazole	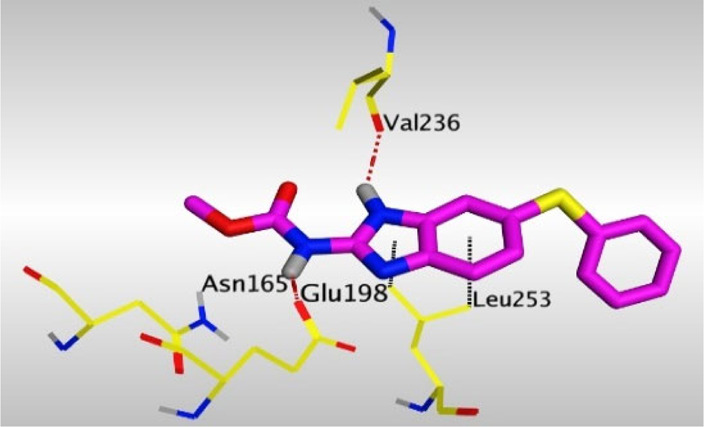	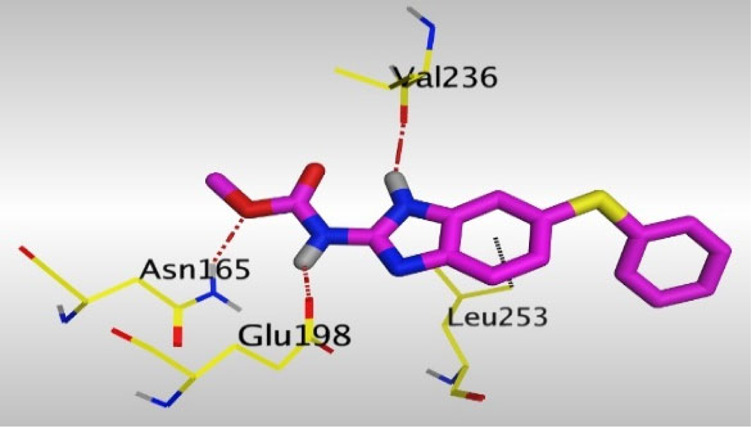
Flubendazole	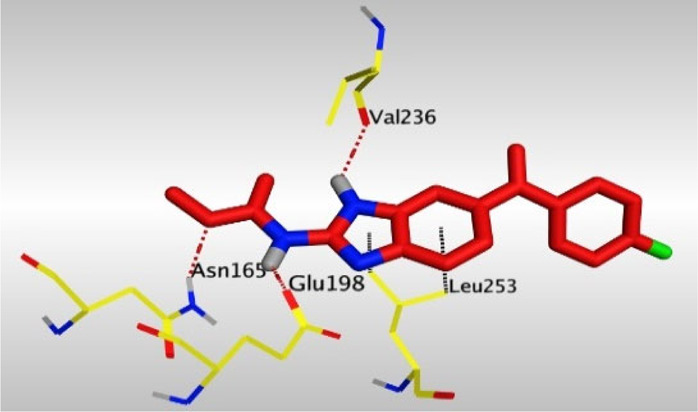	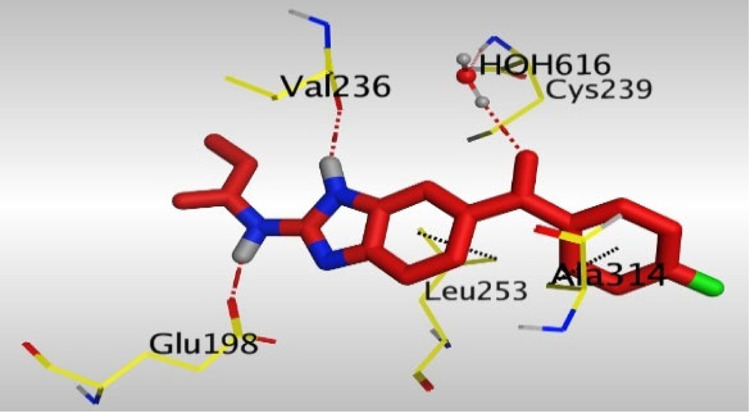
Oxibendazole	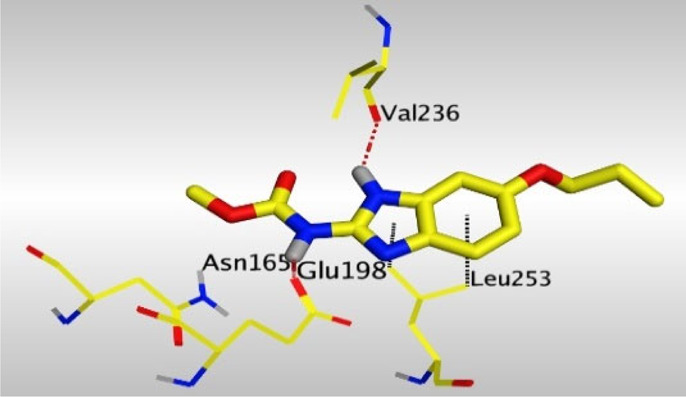	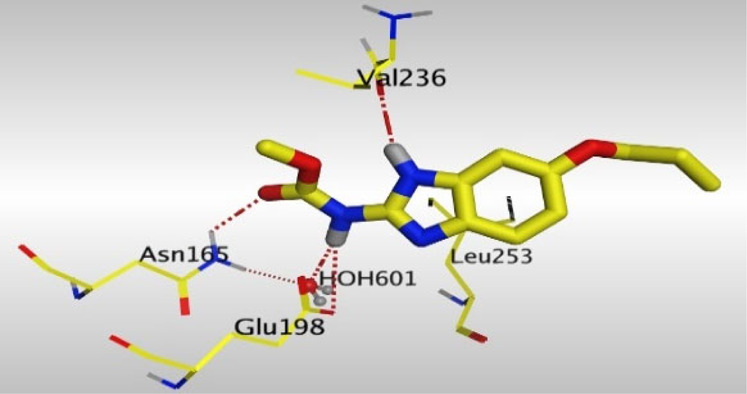
Oxfendazole	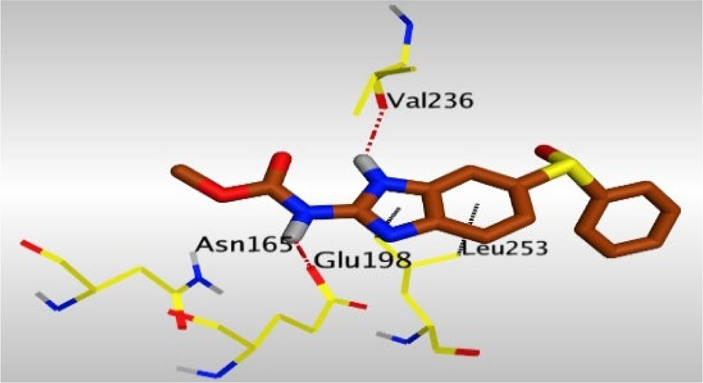	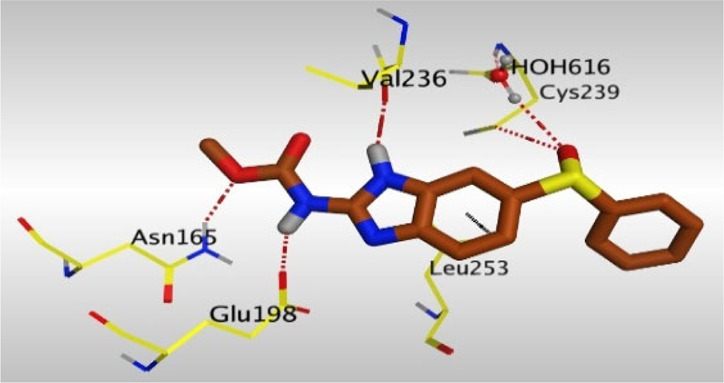
Thiabendazole	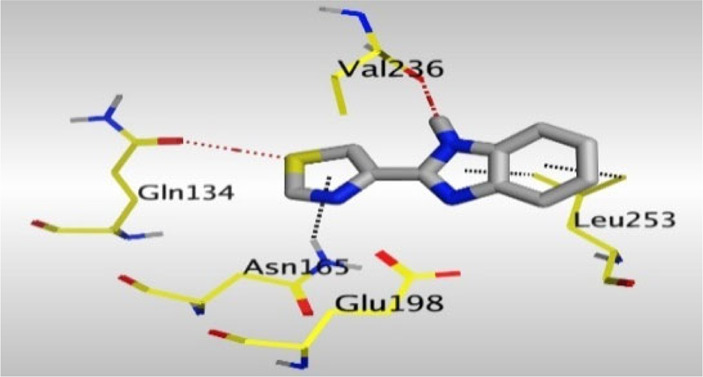	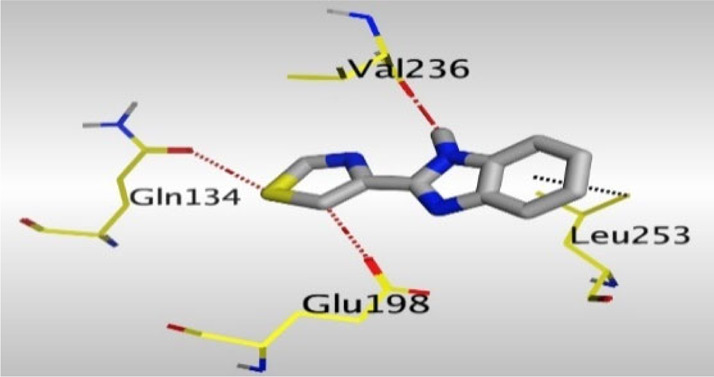
Bendazole	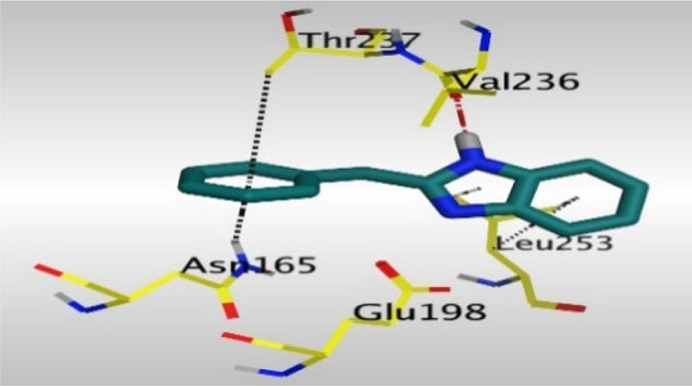	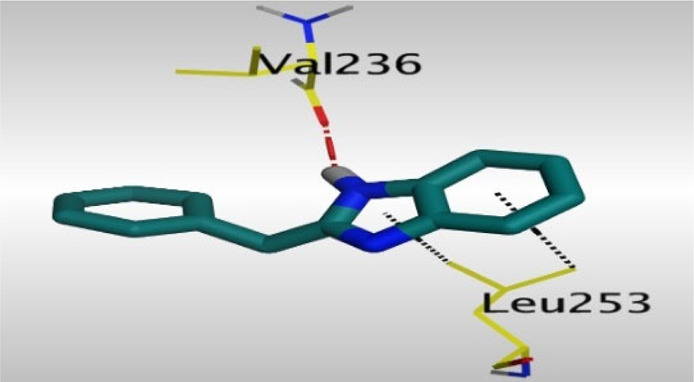

By studying the binding site in both B and D subunits, it was concluded that the crucial amino acids for binding interactions between CBSIs and the two subunits are Glu198 and Val236, besides Asn165 for B subunit and Cys239 for D subunit. The refined NZO was also docked among other studied benzimidazole drugs into subunits B and D to validate the molecular dynamic model used in conducting the current study. For the B subunit, the docked NZO showed almost a fingerprint binding mode as the original co-crystallized one. Interestingly, loss of binding interactions between the oxygen atom of its carbonyl group and Cys239 was also noticed. Furthermore, as expected, the binding interaction between N (3)-benzimidazole and Glu198 was lost in two subunits (B and D) as a result of using the neutral form of refined NZO in contrary to the protonated form of native NZO. The other nine benzimidazole drugs were refined and docked into NZO binding pocket subunits (B and D), respectively. Results revealed that all of the tested drugs, except for Thiabendazole (8) and Bendazole (9) members, showed very similar binding modes to the native NZO due to the great structural similarity. Regarding B subunit, Flubendazole (5) showed a nearly similar fingerprint binding mode compared to the native and docked forms of NZO. Concerning D subunit, surprisingly, Albendazole (2), Ciclobendazole (3), Flubendazole (5), and Oxfendazole (7) showed the binding interaction with Cys239 similar to the native NZO which was lost in the docked NZO itself. Especially Ciclobendazole (3) and Oxfendazole (7) showed a fingerprint binding interaction with Cys239 with the same direct and indirect pathways. It was concluded that Flubendazole (5) showed the highest similarity in binding modes compared to the native NZO in both B and D subunits of protein pocket. Furthermore, Thiabendazole (8) and Bendazole (9) lost some of the binding interactions compared to the native NZO due to the differences in the structure of side chains of their benzimidazole moieties but maintained the binding interactions with both Val236 and Asn165. By reviewing molecular dynamics scoring and other parameters listed in Table 2, all of the tested benzimidazole drugs showed comparable high absolute values for MD scoring and energies (except for Thiabendazole (8) and Bendazole (9) members), and the lowest rmsd_refine values compared to the docked NZO. Mebendazole (1) and Flubendazole (5) showed better scores for binding of its poses inside B and D subunits calculated at −7.36, −7.36, and −7.28, −7.25 respectively compared to the docked poses of NZO at −7.34 and −7.04. Again, Flubendazole (5) is the most promising drug to be repurposed as a colchicine binding site inhibitor compared to the docked NZO and other tested drugs. Moreover, the order of binding affinities for our tested benzimidazole drugs with target Tubulin protein depending on the docking and the calculated physical properties is as follows: Flubendazole (5) > Oxfendazole (7) > NZO (docked) > Mebendazole (1) > Albendazole (2) > Oxibendazole (6) > Fenbendazole (4) > Ciclobendazole (3) > Thiabendazole (8) > Bendazole (9).

**TABLE 2:** Calculated parameters obtained from docking of different benzimidazole drugs and NZO in the binding pockets within subunit B and D of Tubulin protein.

Phys. Prop.	S (subunit B)	S (subunit D)	rmsd_refine	E_conf	E_refine
NZO (docked)	−7.34	—	0.95	0.64	−26.68
	—	−7.04	2.07	−7.66	−27.13
Mebendazole	−7.36	—	1.54	−21.83	−37.08
	—	−7.28	1.40	−20.16	−37.04
Albendazole	−7.23	—	0.97	−53.87	−36.45
	—	−6.86	1.06	−40.74	−30.12
Ciclobendazole	−6.94	—	0.90	62.46	−35.58
	—	−6.78	1.40	63.16	−32.88
Fenbendazole	−7.09	—	1.71	−47.42	−37.47
	—	−7.39	1.22	−46.84	−37.31
Flubendazole	−7.36	—	1.19	−18.75	−36.26
	—	−7.25	1.38	−3.47	−34.07
Oxibendazole	−7.36	—	1.12	−52.05	−38.65
	—	−6.86	1.82	−40.80	−29.96
Oxfendazole	−7.31	—	2.47	−28.07	−38.11
	—	−7.55	2.03	−27.11	−38.19
Thiabendazole	−4.59	—	1.81	43.97	−8.75
	—	−4.68	0.23	39.50	−11.37
Bendazole	−5.44	—	1.12	39.08	−12.60
	—	−5.03	1.60	43.80	−6.76

S, the score of placement of a compound into the binding pocket of protein; rmsd_refine, the root-mean-squared-deviation (RMSD) between the heavy atoms of the predicted pose (after refinement) and those of the crystal structure (before refinement); E_conf, conformer energy in kcal/mol; E_refine, the score of refinement step of ligand conformer.

Another confirmatory tool for validating the binding interactions between the tested ligands and the target protein is through measuring the lengths of H-bonds involved. Table 3 shows H-bonds between our tested benzimidazole drugs compared to the native and docked forms of NZO with the similar amino acids involved in binding interactions. As predicted before, Flubendazole 5) was the best drug exhibiting similar binding mode to the native and docked forms of NZO by forming alone, at B subunit, an H-bond with Asn136 through the oxygen atom of its carbamate moiety calculated at 3.03 Å comparable to 3.19 Å and 3.03 Å formed by the native and docked forms of NZO, respectively. Additionally, it was one of the four drugs reported to interact with Cys239, at D subunit, superior to the docked NZO form by forming an H-bond with the bridging water molecule (H_2_O616) calculated at 3.14 Å which is comparable to the same bond formed at 2.70 Å by the native NZO.

**TABLE 3:** Calculated hydrogen bond length (in Å) between different benzimidazole drugs and NZO and the crucial amino acids at binding sites in subunits B and D of Tubulin protein.

Phys. Prop.	Subunit	O-carbamate	NH-carbamate	N (1)H-benzimidazole	Cys239
					(Direct, indirect)
NZO (native)	B	3.19	2.70	2.94	—
	D	—	2.72	2.92	3.23, 2.70
NZO (docked)	B	3.03	2.95	2.87	—
	D	2.93	2.76	2.80	—
Mebendazole	B	—	2.89	2.91	—
	D	2.99	3.03	2.83	—
Albendazole	B	—	2.85	2.78	—
	D	—	2.71	2.81	3.40, —
Ciclobendazole	B	—	2.99	2.97	—
	D	—	2.92	2.81	3.23, 3.20
Fenbendazole	B	—	2.96	2.86	—
	D	2.95	3.03	2.81	—
Flubendazole	B	3.03	2.93	2.92	—
	D	—	2.79	2.84	—, 3.14
Oxibendazole	B	—	2.89	2.82	—
	D	2.87	3.53	3.00	—
Oxfendazole	B	—	2.97	2.87	—
	D	2.95	3.04	2.81	3.30, 3.38
Thiabendazole	B	—	—	2.74	—
	D	—	—	2.66	—
Bendazole	B	—	—	2.72	—
	D	—	—	2.78	—

By representing the 3D filling positions of our tested compounds inside the deep protein pockets of two subunits B and D compared to the co-crystallized inhibitor, we observed a very close similarity between all in each subunit confirming the same binding mode and structural similarity between them as shown in Table 4.

**TABLE 4:** The 3D positioning of different forms tested benzimidazole drugs inside the deep binding pocket of NZO within Tubulin subunit B and D (PDB: 5CA1) alongside with the solved NZO complex (Native) and the docked complex (Docked).

Drug	Protein (5CA1) subunit B	Protein (5CA1) subunit D
NZO (native)	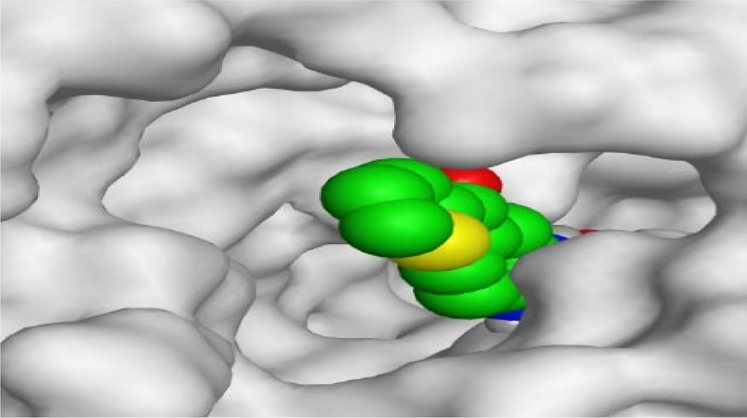	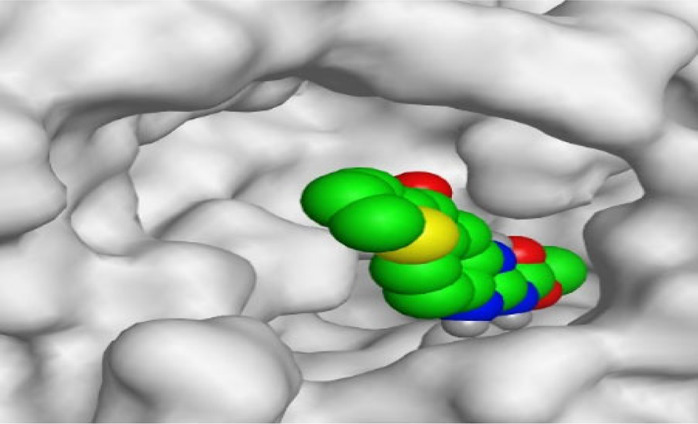
NZO (docked)	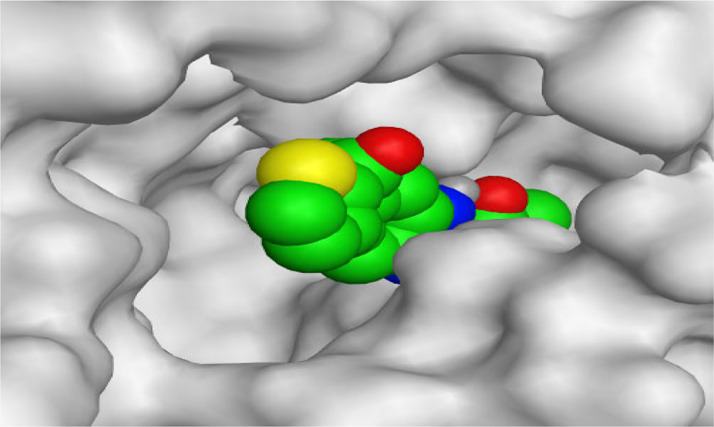	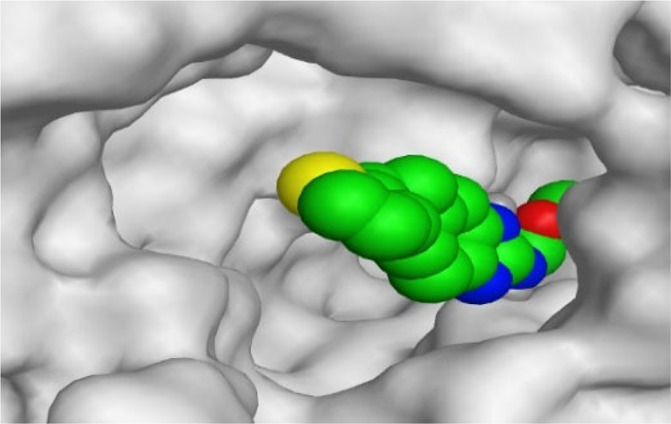
Mebendazole	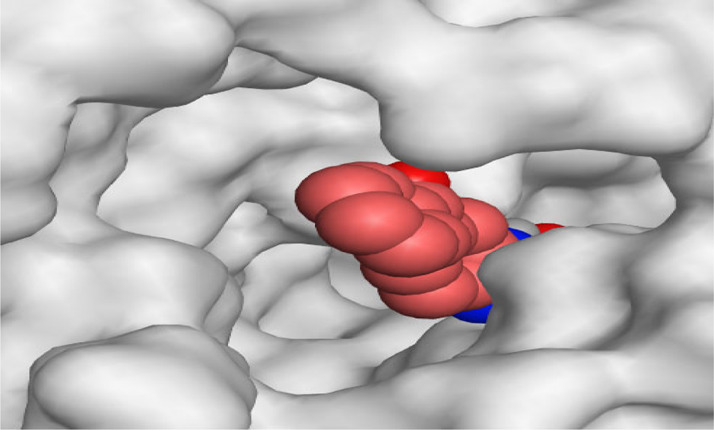	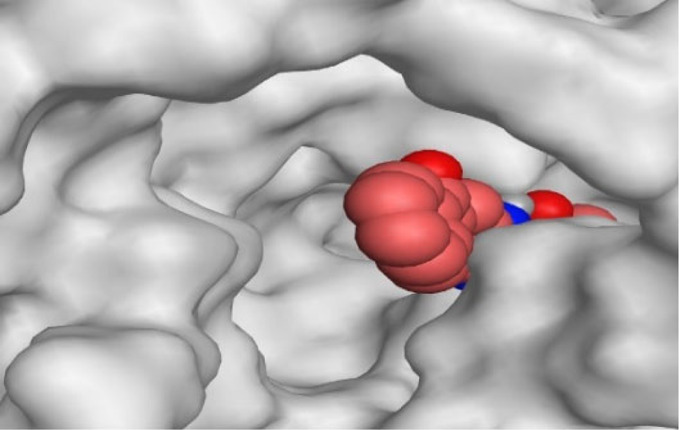
Albendazole	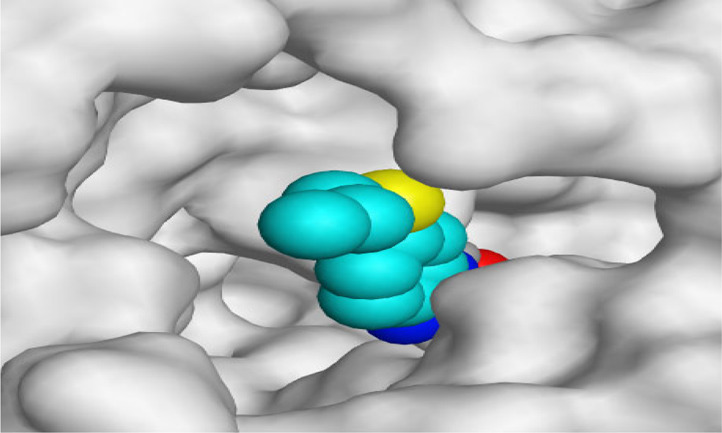	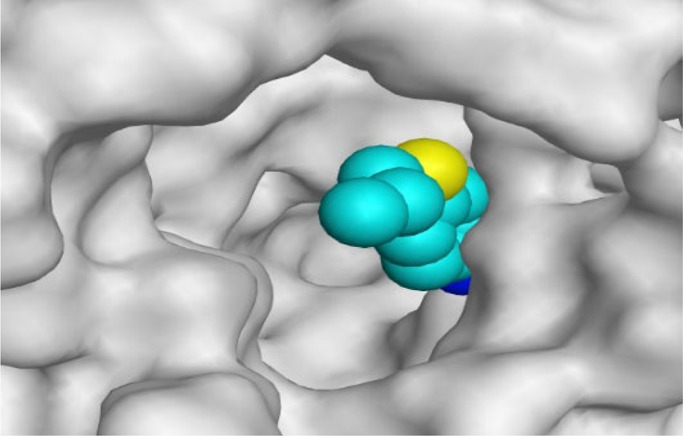
Ciclobendazole	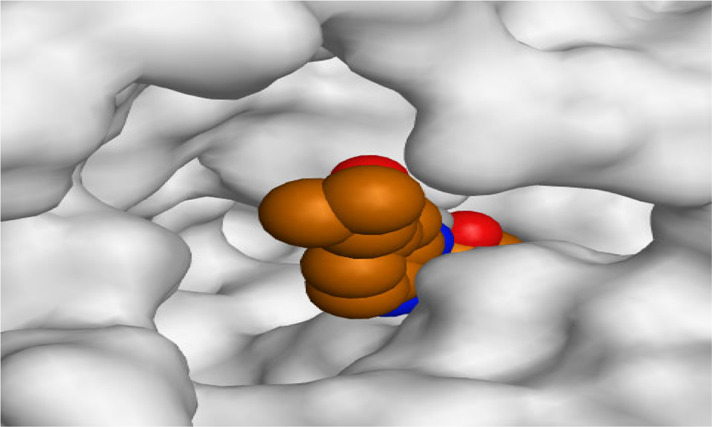	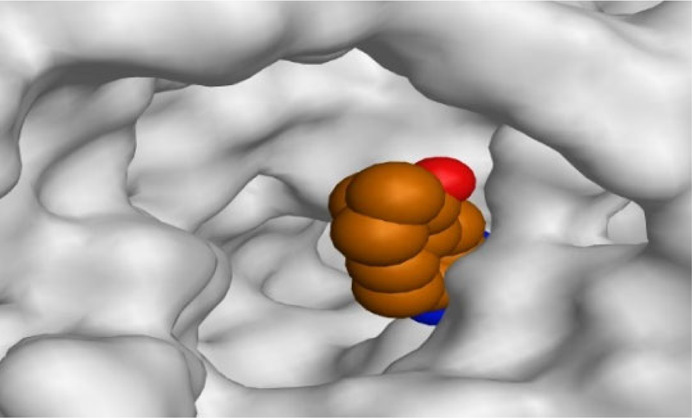
Fenbendazole	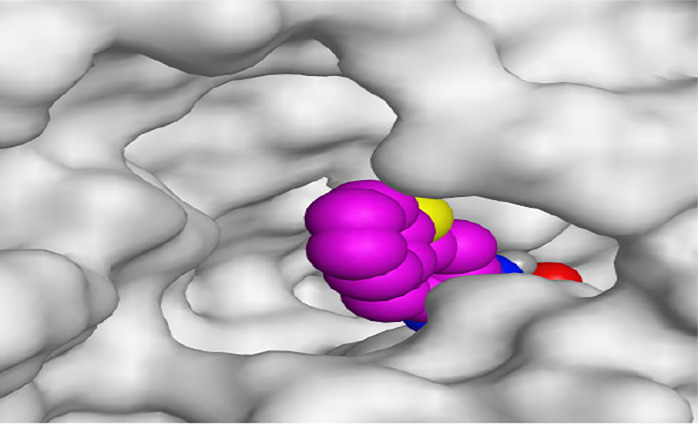	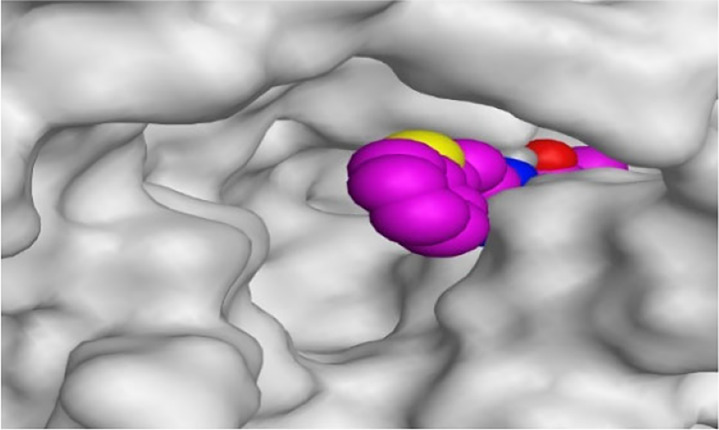
Flubendazole	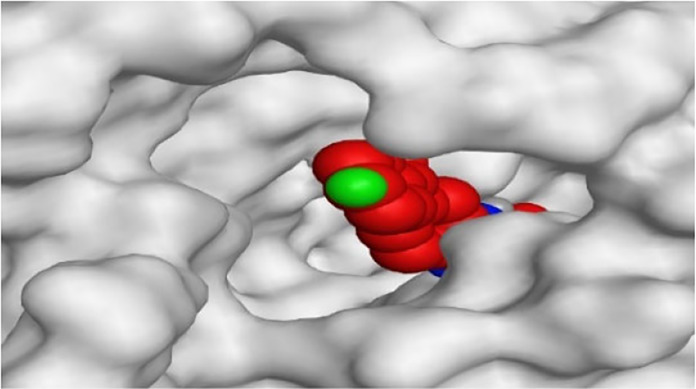	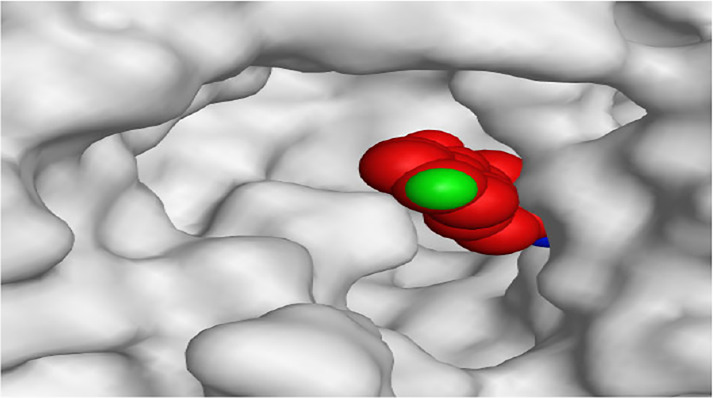
Oxibendazole	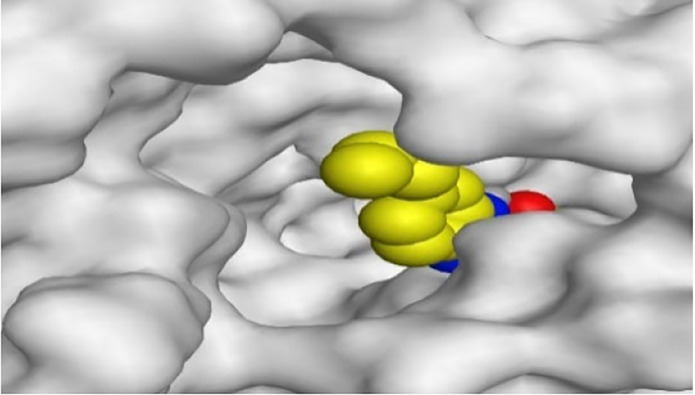	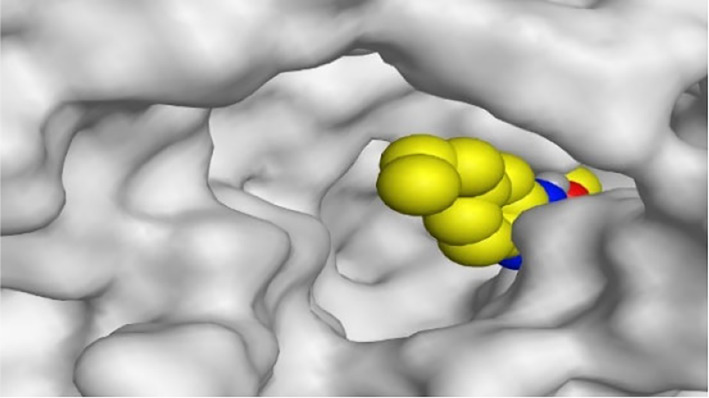
Oxfendazole	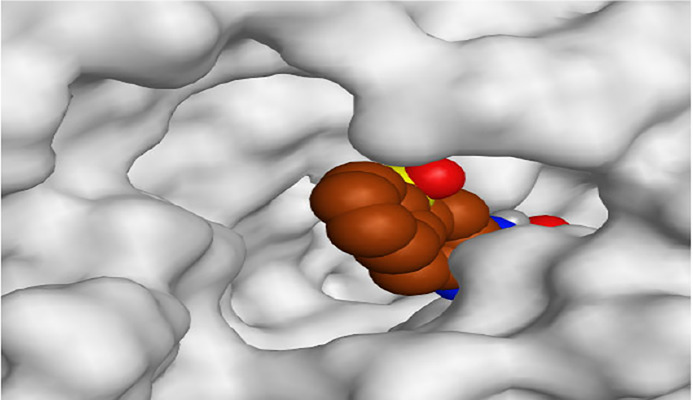	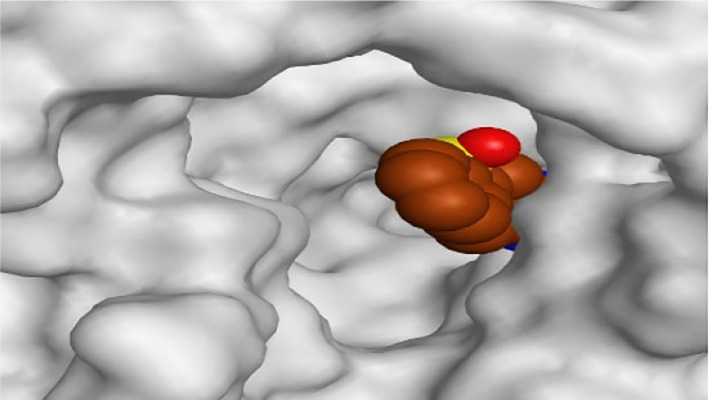
Thiabendazole	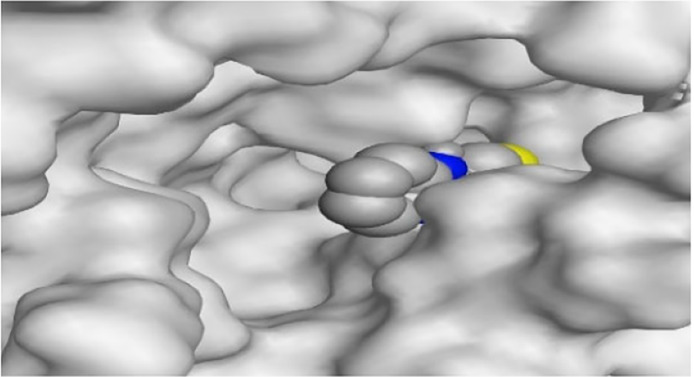	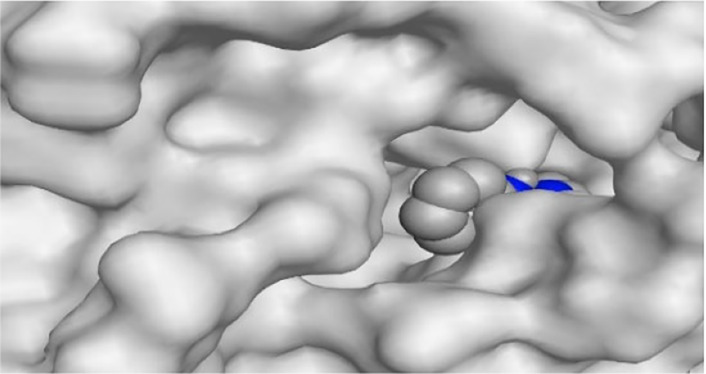
Bendazole	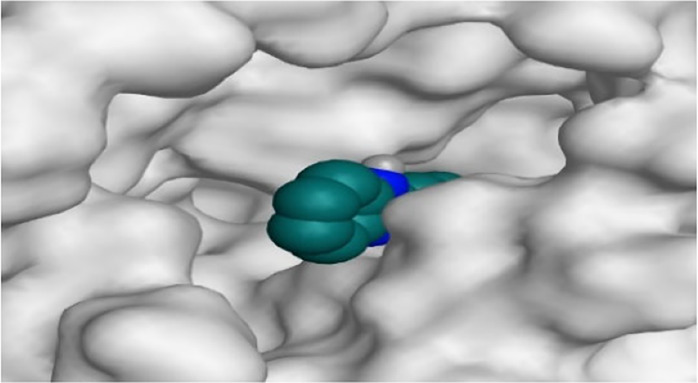	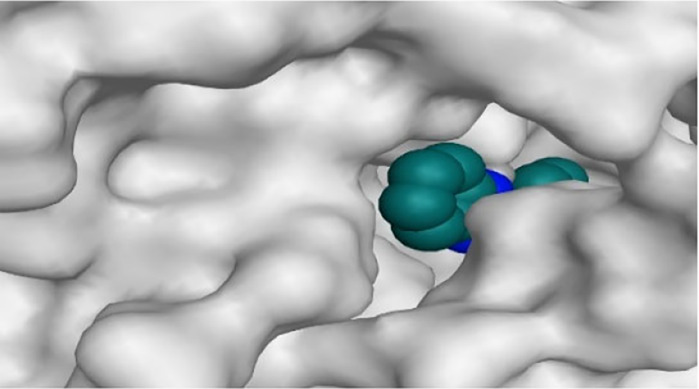

Moreover, the large size of the two pockets of subunits B and D, especially D subunit, gives us an idea about the possible drug modifications especially at the side chain of thiophene ring of NZO to obtain larger compounds that can occupy the binding pockets more efficiently maintaining the same essential binding interactions with the crucial amino acids and at the same time forming extra binding ones for better inhibition.

It is well known that outermost electrons are those involved in the binding interaction between a ligand and target protein, we therefore, computed the electron density of the highest occupied molecular orbital (HOMO), the lowest unoccupied molecular orbital (LUMO), and the molecular electrostatic potential (MEP) map. Figures of the electron density distribution of HOMO, LUMO, and MEP are depicted in Table 5.

**TABLE 5:** The charge density of the highest occupied molecular orbital (HOMO), the lowest unoccupied molecular orbital (LUMO), and the molecular electrostatic potential (MEP) map of studied compounds. Red and blue color codes represent the most electronegative and electropositive density, respectively. B3LYP/6-311+G* level of theory was employed to compute molecular orbital energies.

	HOMO	LUMO	MEP
NZO (docked)	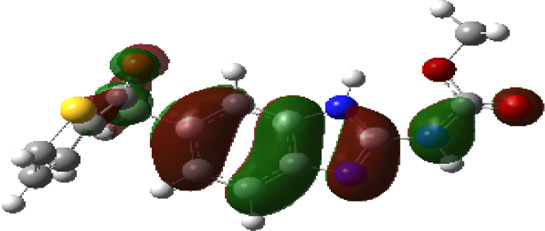	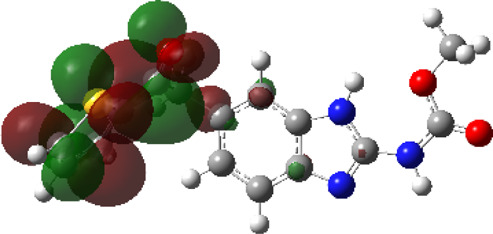	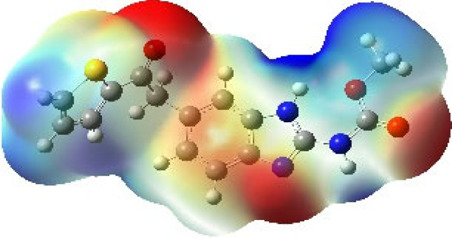
Mebendazole	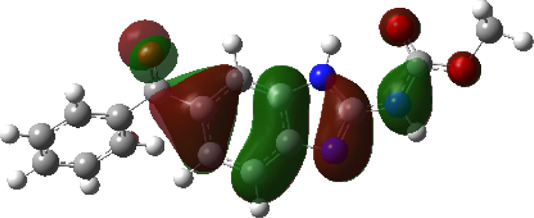	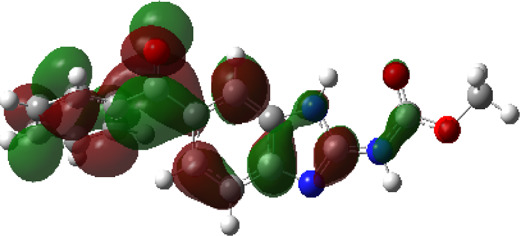	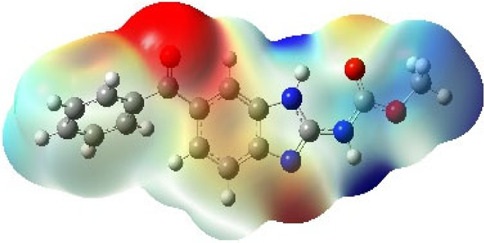
Albendazole	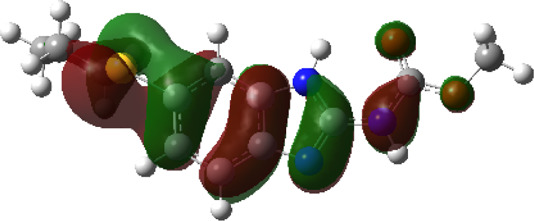	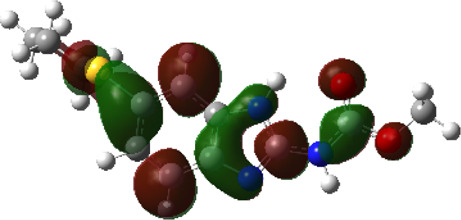	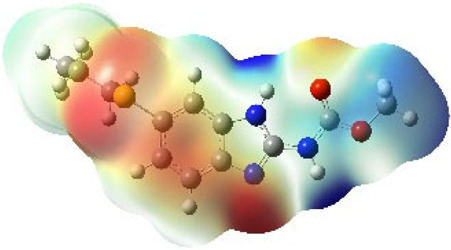
Ciclobendazole	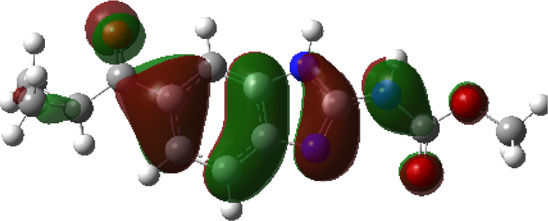	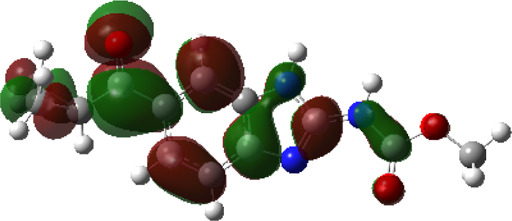	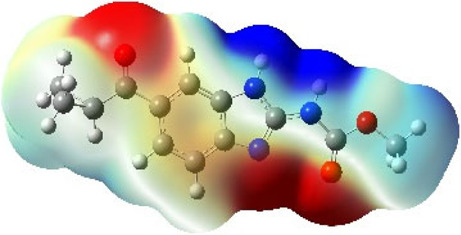
Fenbendazole	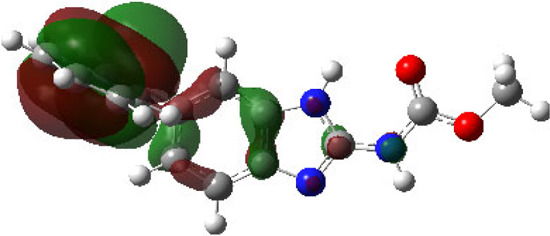	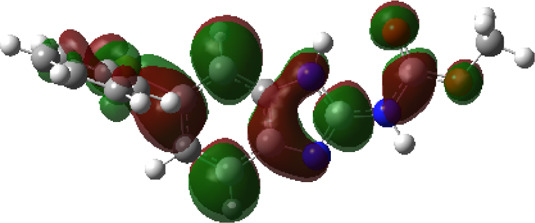	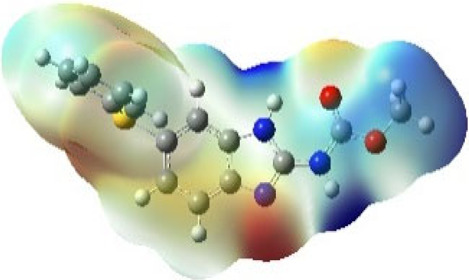
Flubendazole	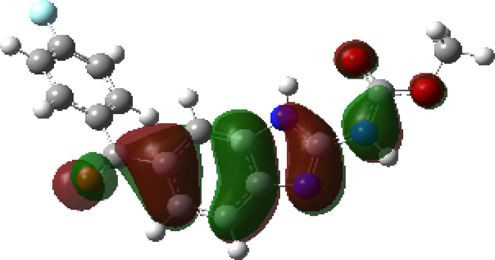	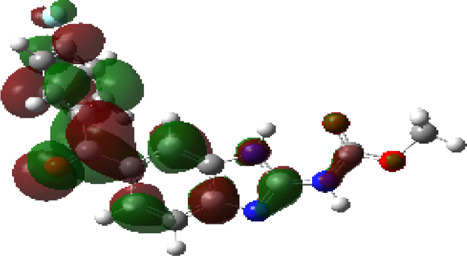	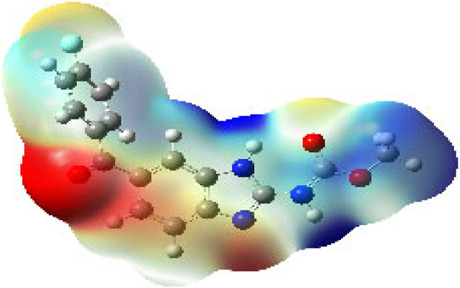
Oxibendazole	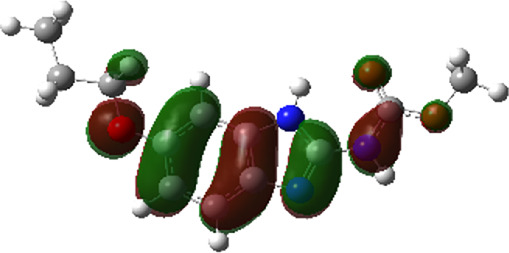	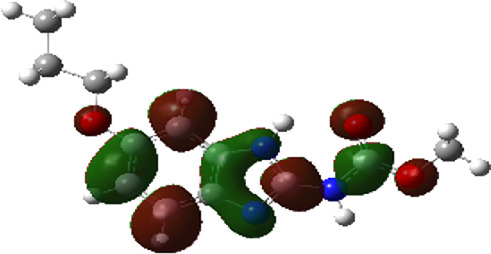	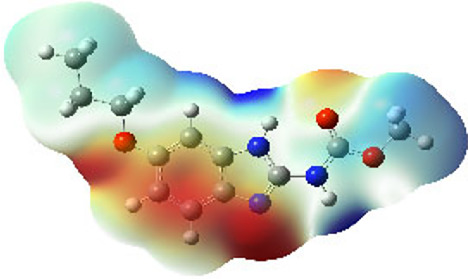
Oxfendazole	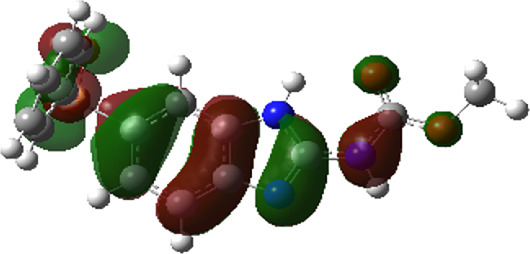	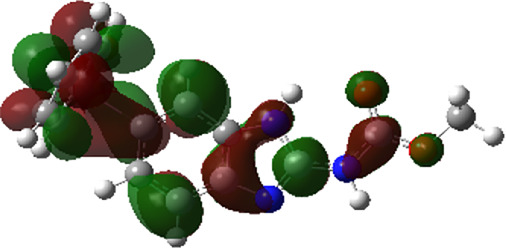	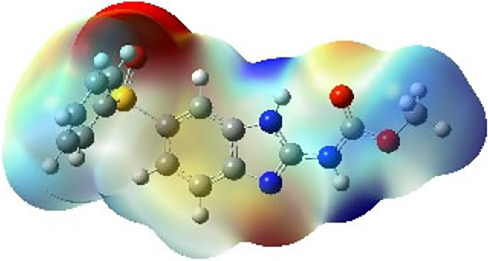
Thiabendazole	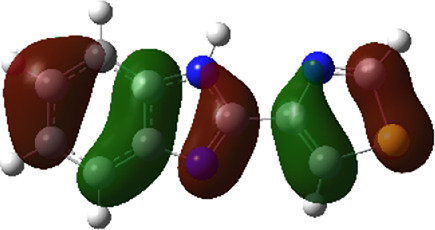	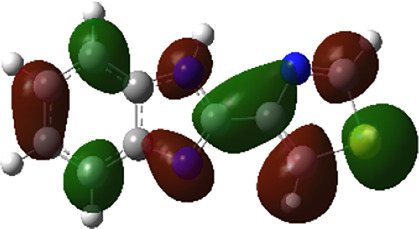	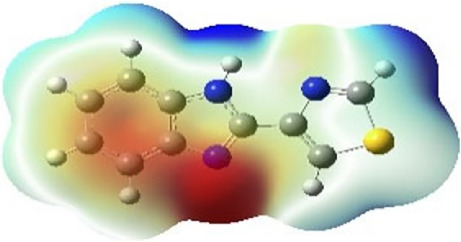
Bendazole	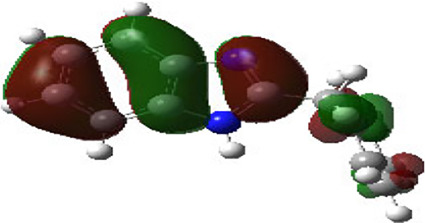	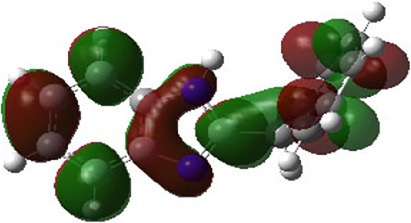	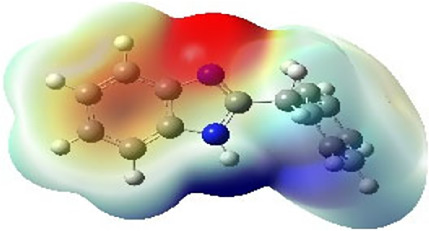

The electron density of HOMO in NZO was found to be localized mainly on benzimidazole moiety, while the electron density of LUMO was solely localized on the thiophene side chain. The electron density of HOMO and LUMO of all other studied drugs are delocalized except for Mebendazole (1), Fenbendazole (4), and Flubendazole (5) in the case of HOMO and Oxibendazole (6) in case of LUMO. The electron density of HOMO of Mebendazole (1) and Flubendazole (5) is quite similar to that of NZO which is consistent with the calculated binding affinities [Flubendazole (5) > Oxfendazole (7) > NZO (docked) > Mebendazole (1)]. Despite of this consistency, the binding affinity does not solely depend on the energies and the electronic density distribution of HOMO and LUMO, but also on other determinants such as hydrogen bonding, electrostatic interactions, hydrophobic and Van der Waals forces and presence of clusters of water. We also noted that the electrons of HOMO are localized mainly on the benzene moiety and the aromatic side chain of Fenbendazole 4) in a different pattern to that observed with NZO.

The MEP maps of all studied compounds revealed that one of benzimidazole nitrogen acts as an electron donating site capable of forming H-bond with amino acids in colchicine binding sites. Whereas the other benzimidazolyl nitrogen is electrophilic moiety acting as H-donor group. Interestingly, the MEP map of Mebendazole (1), Albendazole (2) and Flubendazole (5) are quite similar which is also consistent with the calculated binding affinities, where Flubendazole (5) > Oxfendazole (7) > NZO (docked) > Mebendazole (1) > Albendazole (2) > Oxibendazole (6) > Fenbendazole (4) > Ciclobendazole (3) > Thiabendazole (8) > Bendazole (9). Lack of 5 (6) substitution on the benzimidazole pharmacophoric group leads to a significant change in the MEP of Thiabendazole and Bendazole. Weaker binding affinities of these two drugs can be ascribed to the change in electronic configuration due to the absence of 5 (6) substitution.

## Conclusion

Among the nine tested members of benzimidazole anthelmintic drugs, Flubendazole (5) exhibits the most similar binding interactions, scoring, electron density distribution, and electrostatic map to that reported for NZO in tubulin protein (PDB: 5CA1). This suggests that Flubendazole (5) would be the most active member exerting antitumor activity mainly at CBS. At the same time, more structural modifications are required for FDA approved benzimidazole anthelmintic drugs and the original NZO inhibitor to obtain larger compounds with better fitting and binding modes inside the two large pockets of subunits B and D respectively.

## Data Availability

The original contributions presented in the study are included in the article/Supplementary Material, further inquiries can be directed to the corresponding authors.
