# Supplementary material for: Revisiting Activity of Some Nocodazole Analogues as a Potential Anticancer Drugs Using Molecular Docking and DFT Calculations
Source: Front Chem. 2021 Mar 24;9:628398. doi: 10.3389/fchem.2021.628398 (PMC8024586; doi:10.3389/fchem.2021.628398)
Supplement: Supplementary file 1 [file table1.docx]

**Supplementary data**

**Table 1.** The 2D view of binding interactions between tested benzimidazole drugs and Nocodazole-binding pocket within Tubulin subunit B and D (PDB: 5CA1) alongside with the solved Nocodazole (NZO) complex (Native) and the docked complex (Docked).

**
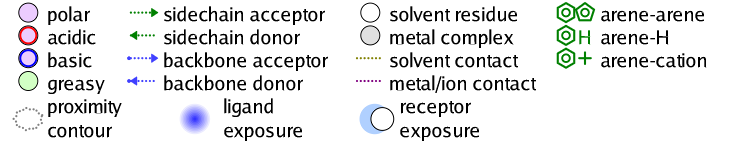
**

| **Drug** | **Protein (5CA1) subunit B** | **Protein (5CA1) subunit D** |
| --- | --- | --- |
| **NZO (Native)** | 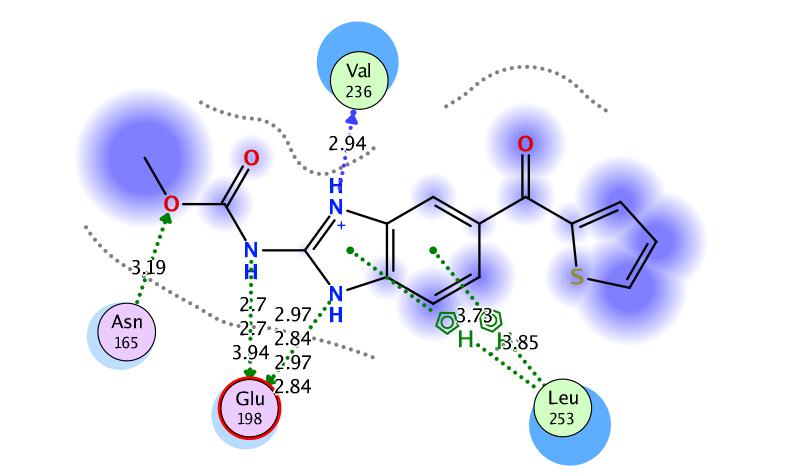 | 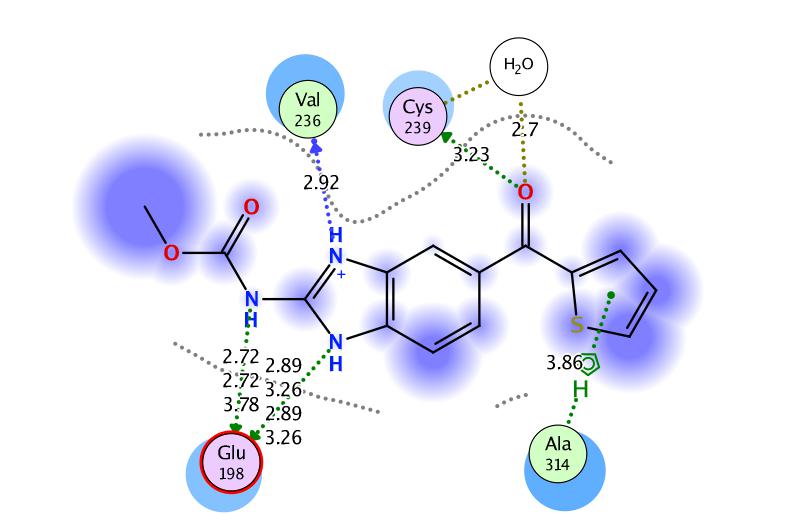 |
| **NZO (Docked)** | 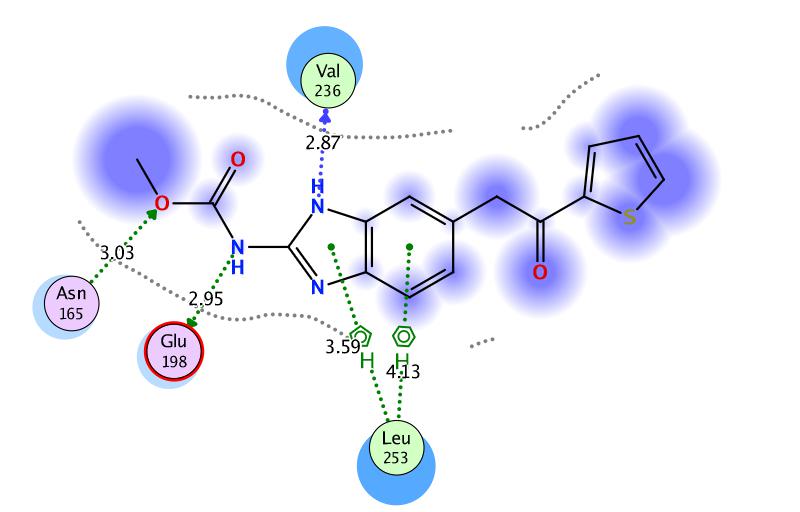 | 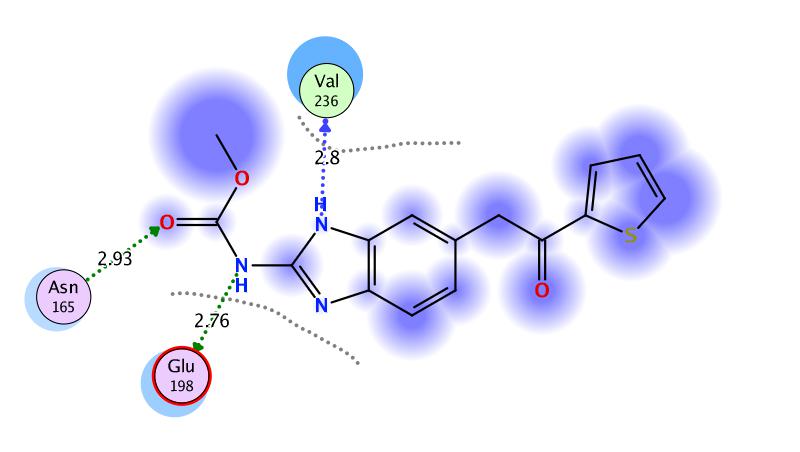 |
| **Mebendazole** | 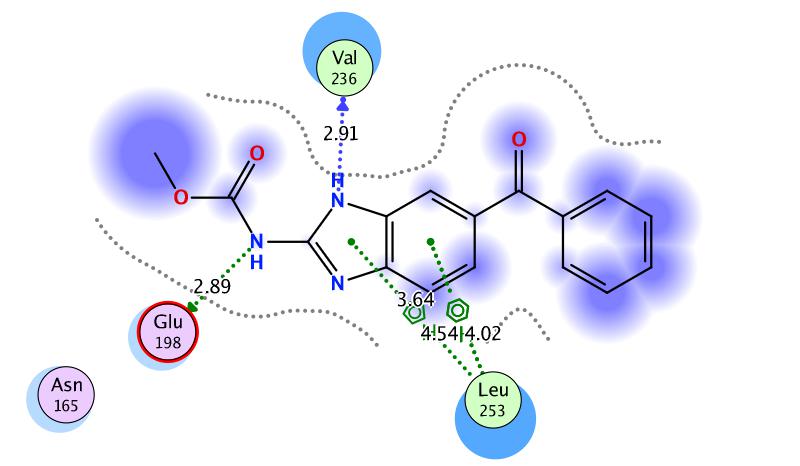 | 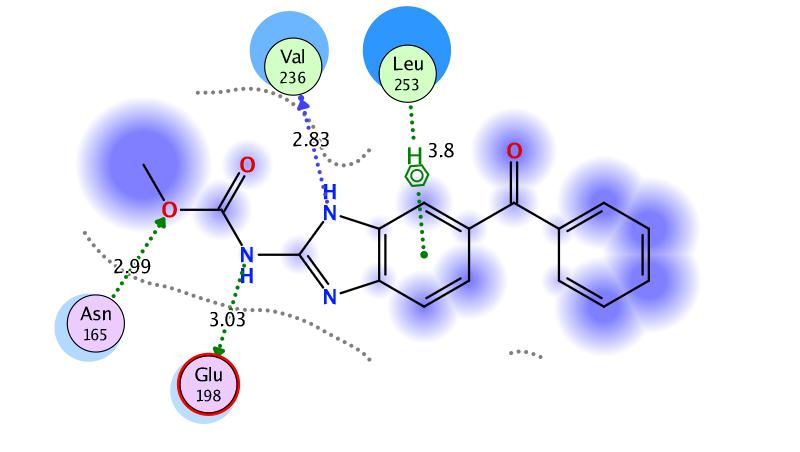 |
| **Albendazole** | 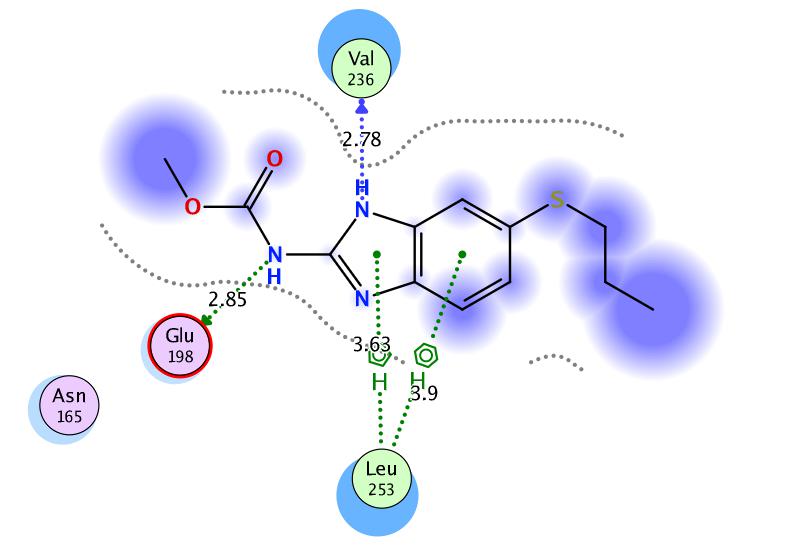 | 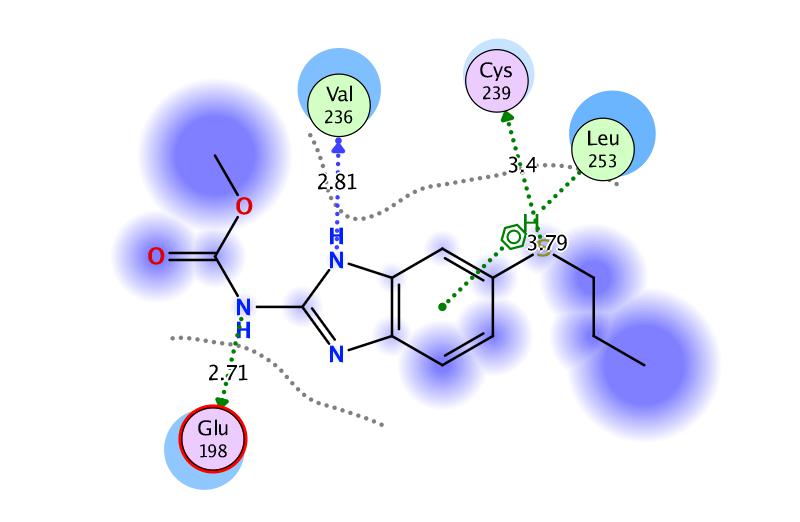 |
| **Ciclobendazole** | 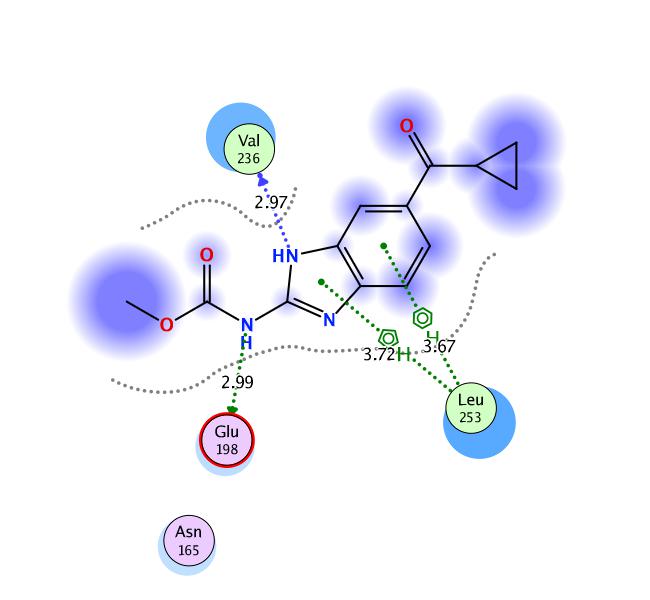 | 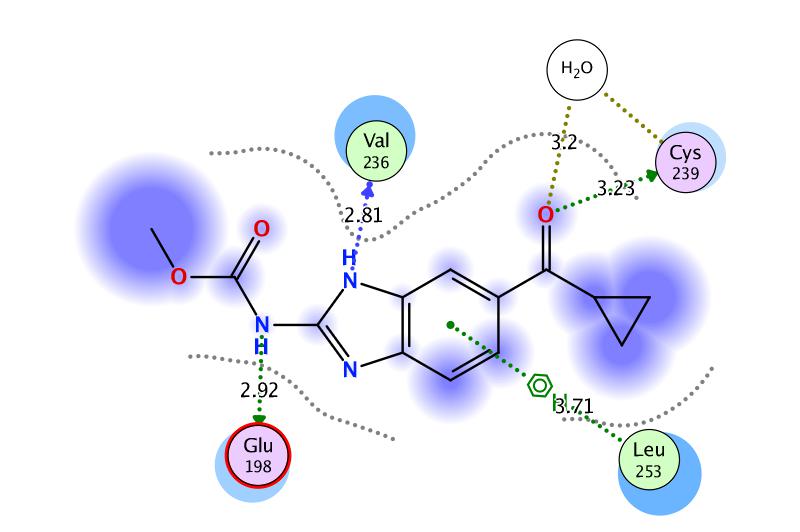 |
| **Fenbendazole** | 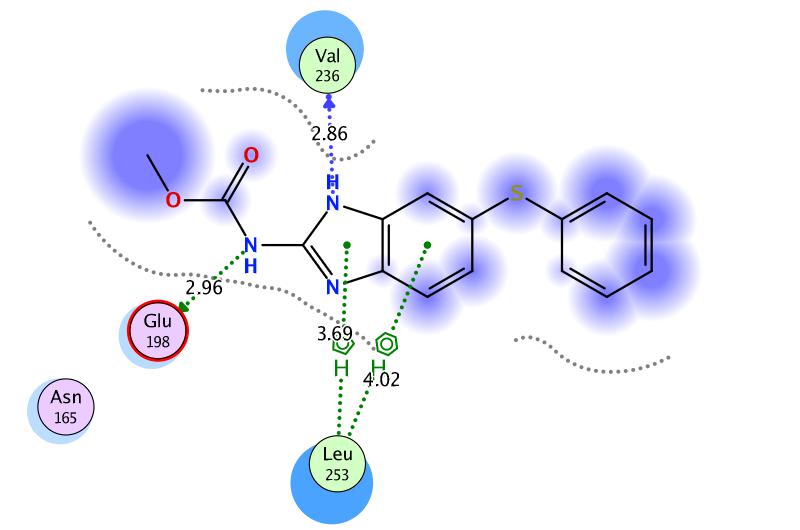 | 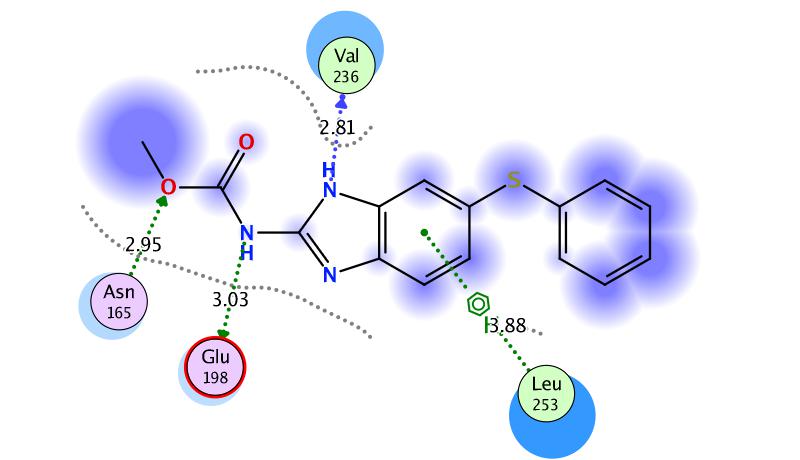 |
| **Flubendazole** | 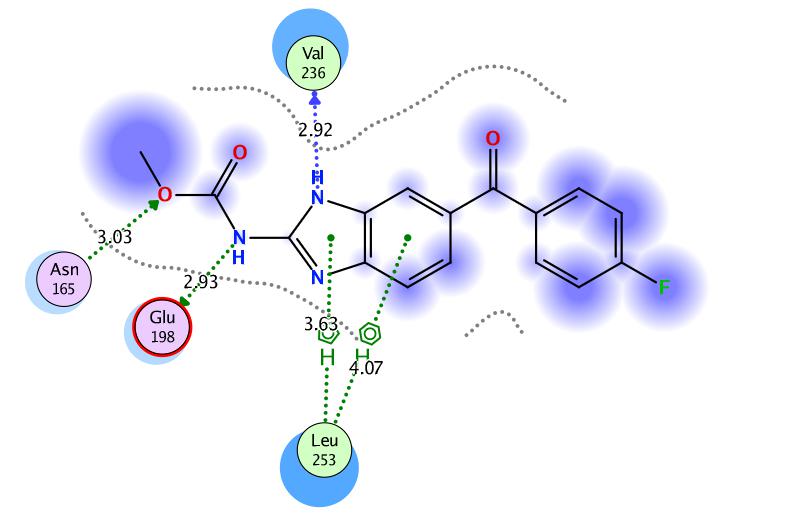 | 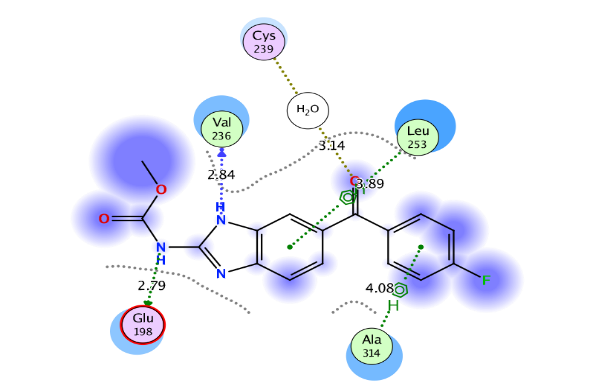 |
| **Oxibendazole** | 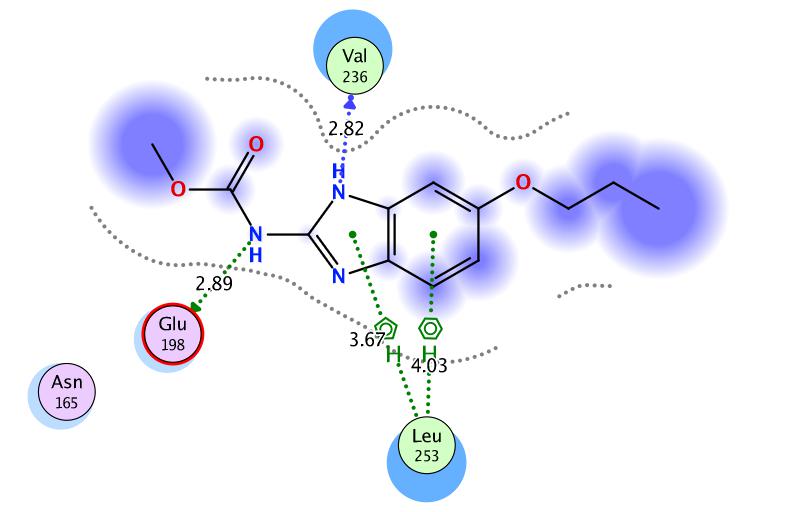 | 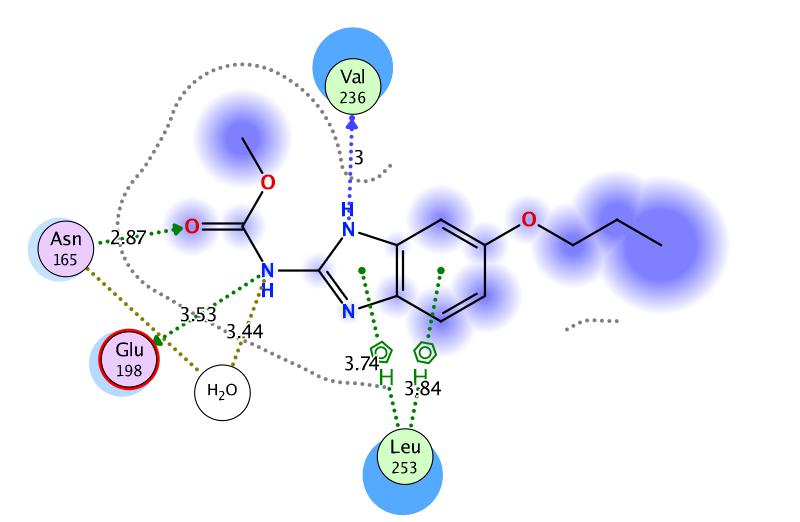 |
| **Oxfendazole** | 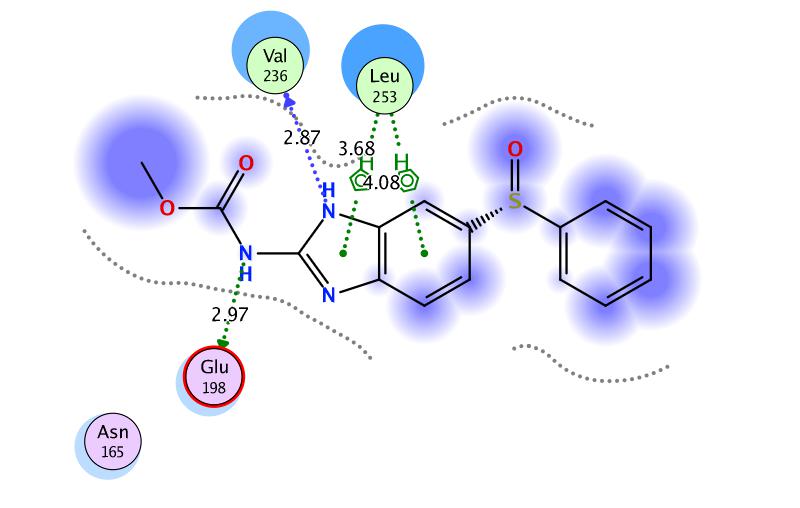 | 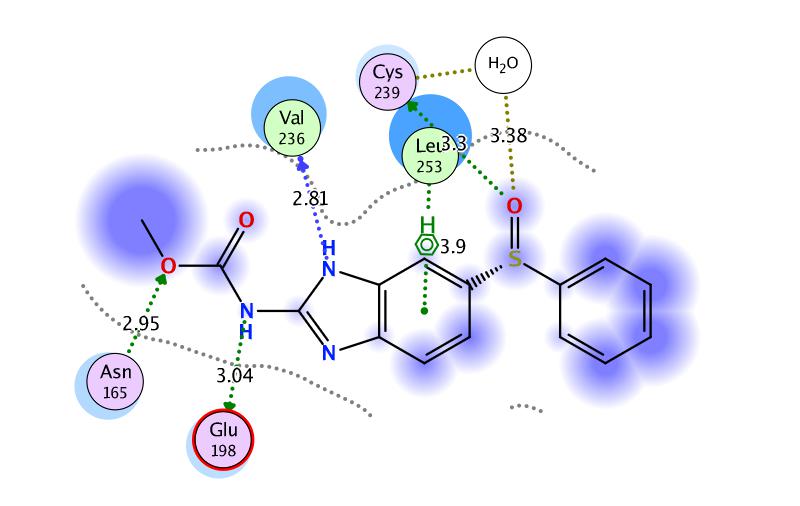 |
| **Thiabendazole** | 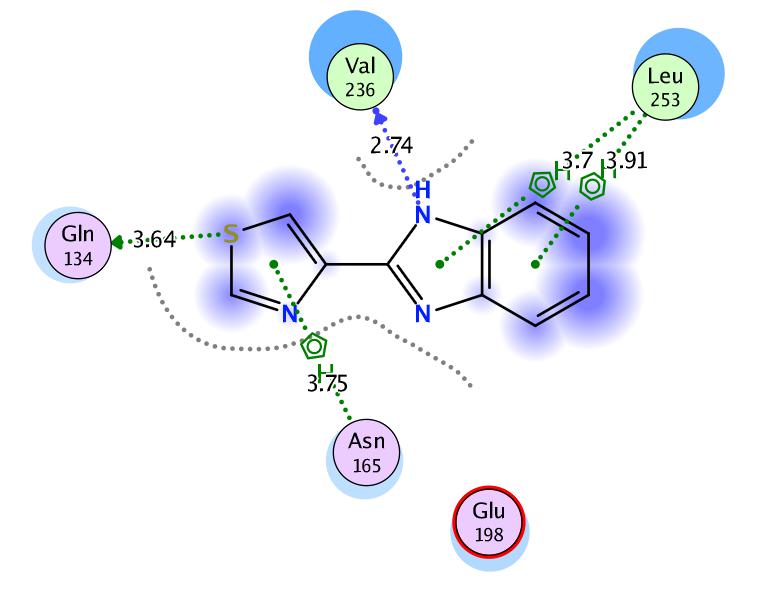 | 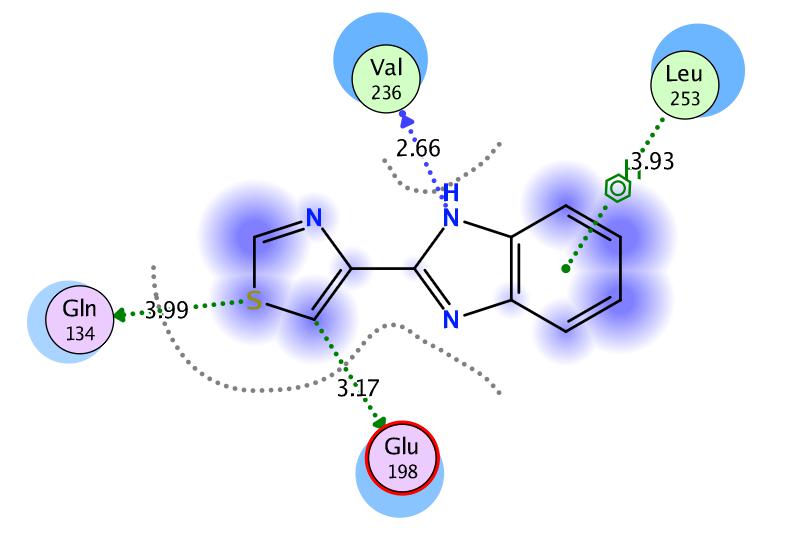 |
| **Bendazole** | 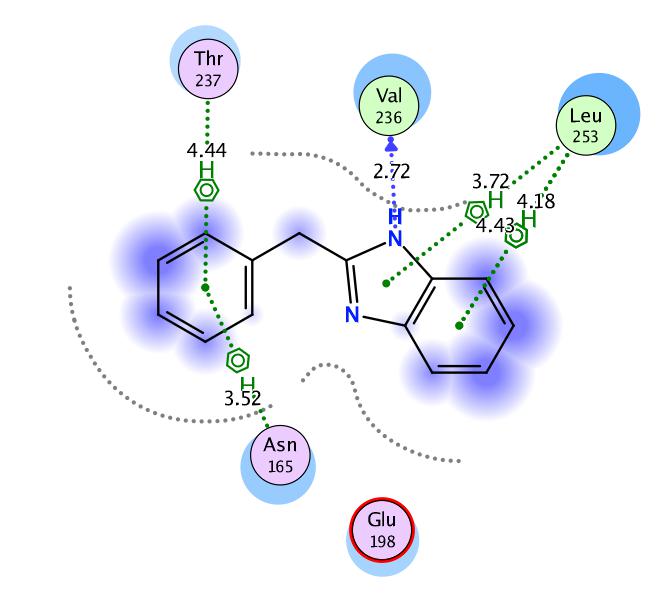 | 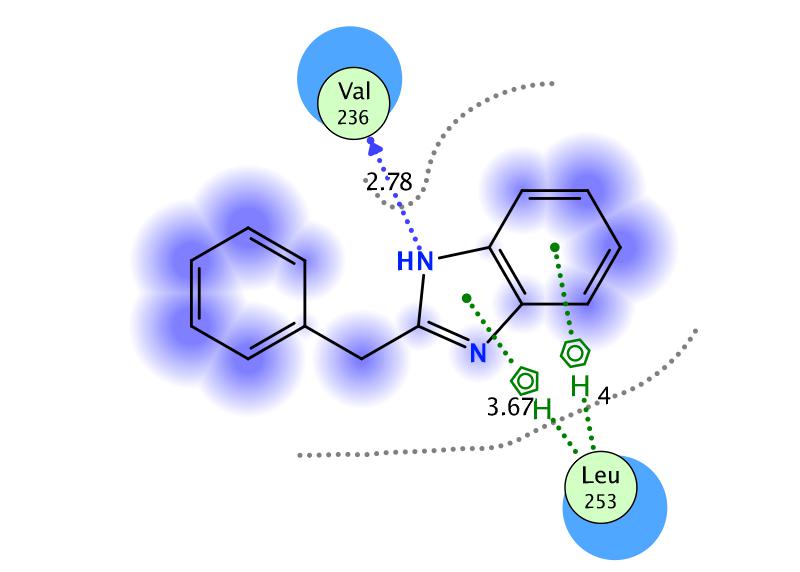 |
